# An ecophysiological approach to improving health outcomes of ornamental shrimp during aquarium trade and transportation

**DOI:** 10.1093/conphys/coag046

**Published:** 2026-07-11

**Authors:** Sancha Conway Holroyd, John I Spicer, Robert P Ellis, Lucy M Turner

**Affiliations:** Bioscience, The University of Exeter, Prince of Wales Rd, Exeter EX4 4PS, UK; School of Biological and Marine Science, University of Plymouth, Plymouth, Devon PL48AA, UK; School of Biological and Marine Science, University of Plymouth, Plymouth, Devon PL48AA, UK; Bioscience, The University of Exeter, Prince of Wales Rd, Exeter EX4 4PS, UK; School of Biological and Marine Science, University of Plymouth, Plymouth, Devon PL48AA, UK

**Keywords:** Aquarium trade, ecophysiology, health, ornamentals, shrimp

## Abstract

Ecophysiology examines the interactions between an organism’s physiology and its environment. It seeks to understand how an organism functions and adapts under changing conditions. This has direct applications to live-trade aquaculture sectors, including the ornamental aquarium trade, where traded animals experience a variety of changing, and often stressful, environmental conditions that can threaten their health and survival. This review proposes a role for ecophysiology in managing organismal health, using Hatch (1962) theoretical framework of disease and dysfunction as a foundation for defining and distinguishing health. We focus on tropical (marine and freshwater) ornamental shrimp as an exemplar group to highlight critical research gaps and demonstrate how ecophysiological principles can translate into improved health management strategies for animal during trade and transport. Specifically, we (i) show how ecophysiology can be used to inform an operational definition of health, using Hatch (1962) scheme as originally applied to occupational health, (ii) consider how ecophysiological approaches could be applied to ornamental management to improve health outcomes for traded animals, (iii) review current knowledge of how key physico-chemical parameters (temperature, dissolved oxygen, salinity, carbonate chemistry and nitrogenous waste) affect the physiology of tropical ornamental shrimp and (iv) identify knowledge gaps and research opportunities that will inform evidence-based management of these species. We conclude that integrating species-specific ecophysiological knowledge into the ornamental aquarium trade offers a critical pathway towards improving evidence-based health management. By moving beyond disease-centric assessments and addressing fundamental ecophysiological knowledge gaps, we can improve health outcomes and enhance the sustainability and economic viability of this vast global industry. These findings are intended to be of value to researchers across ecophysiology, conservation biology and aquatic animal health, as well as to industry stakeholders responsible for husbandry and transport protocols.

##  Abbreviations

**CO**_**2**_
: carbon dioxide
**CO**_**3**_^**2−**^
: carbonate
**H**_**2**_ **CO**_**3**_
: carbonic acid
**MO**_**2**_
: rate of oxygen consumption
**NH**_**3**_
: ammonia
**O**_**2**_
: oxygen
***P*CO**_**2**_
: partial pressure of dissolved carbon dioxide
pH
: measure of hydrogen ion concentration
***P*O**_**2**_
: partial pressure of dissolved oxygen

## Introduction

### What is the aquarium trade?

The ‘marine’ and ‘tropical’ ornamental industries encompass the international trade of tropical saltwater and freshwater species for home and public aquariums. Although separate industries, they are often placed under the umbrella term of the ‘aquarium trade’ (Palmtag, 2017; Borges *et al.*, 2022). Small-to-medium-scale fisheries operating across the Indo-Pacific, Neotropical, Oceanic and Afrotropical regions drive the global supply of aquarium livestock (Wabnitz *et al.*, 2003; Palmtag, 2017; Rhyne *et al.*, 2017; Evers *et al.*, 2019). While accurately quantifying the scale and value of this global trade is difficult, due to poor catch data and incomplete tracking of export and import activities (Wabnitz *et al.*, 2003; Smith *et al.*, 2008), it is clearly of huge economic value. Estimations value the global aquarium trade between $15 and $30 billion USD (Penning *et al.*, 2009; Food and Agriculture Organisation of the United Nations, 2021), with freshwater dominating the market with respect to the number of species and individuals traded (Evers *et al.*, 2019). Despite freshwater dominance in numbers of individuals, many marine species possess a greater individual retail value, acquiring around an additional 15% of the market value depending on species identity (Dey, 2016). The production and trade of animals for the aquarium trade is a rapidly growing sector within aquaculture (Tlusty, 2002). Of the >5300 freshwater species sold globally, more than 90% are captive bred individuals (Raghavan *et al.*, 2013). For marine species, the opposite is true with ~90% of the market consisting of wild caught specimens (Chapman *et al.*, 1997; Dominguez and Botella, 2014). That said, the landscape is shifting with increasing success in marine captive breeding efforts (Olivotto *et al.*, 2011). For the marine ornamental trade, recent estimations place its value at $2.15 billion, with around 55 million marine organisms sold globally each year (Watson *et al.*, 2023), across more than 2000 species of fish, invertebrates and coral (Wabnitz *et al.*, 2003; Livengood and Chapman, 2007; Palmtag, 2017; Rhyne *et al.*, 2017; Raja *et al.*, 2019). Growing demand and a recent shift in hobbyist interests, from fish-only tanks to emulating micro reef environments (Rhyne *et al.*, 2009), has increased the numbers of species traded.

### Ornamental shrimp

**Figure 1:**
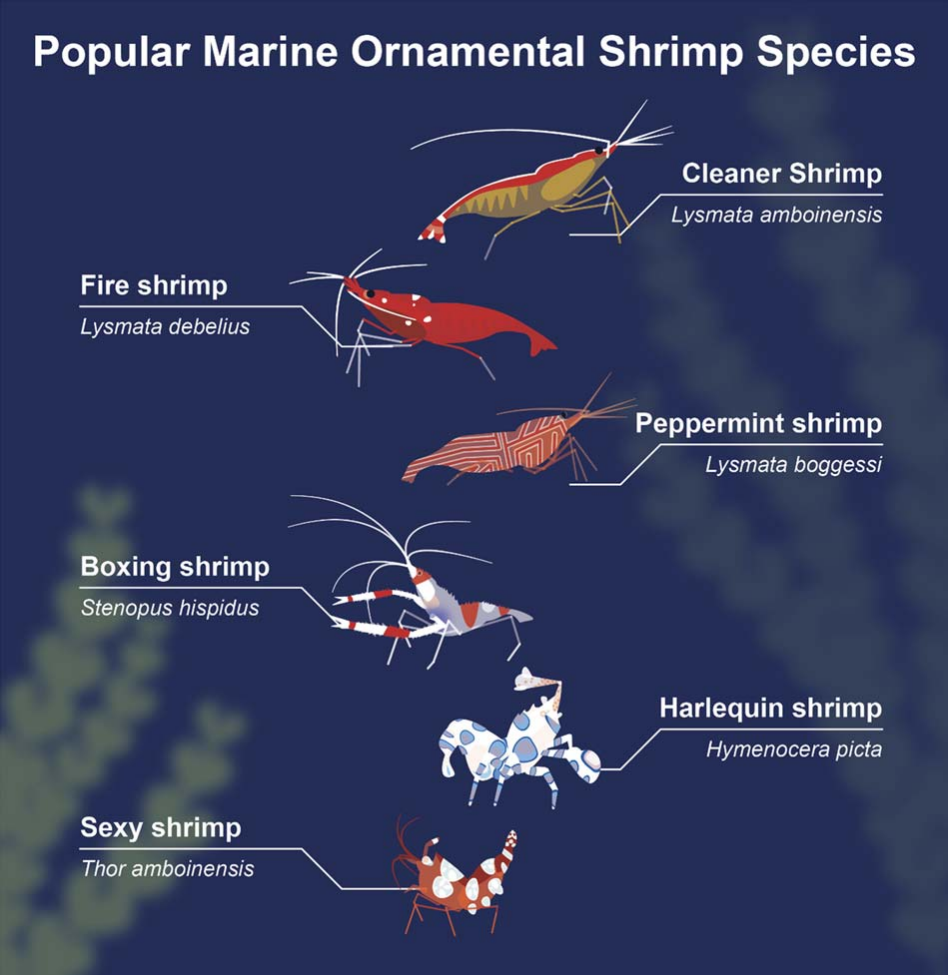
Illustration of the morphological features and colouration patterns of six species of marine ornamental shrimp, commonly traded in the aquarium trade. From top to bottom: *L. amboinensis* (cleaner shrimp, maximum body length = ~2.5–3 inches), *L. debelius* (fire shrimp, maximum body length = ~2.5–3 inches), *L. boggessi* (peppermint shrimp, maximum body length = ~2 inches), *S. hispidus* (boxing shrimp, maximum body length = ~3–4 inches), *H. picta* (harlequin shrimp, maximum body size = ~1–2 inches) and *T. amboinensis* (sexy shrimp, maximum body size = ~0.5–1 inch). Size of illustrated shrimp is not to scale. Figure by Hannah Whitman, Science Animation Studio.

Within the aquarium trade, ornamental shrimp make up a highly popular category of traded organisms. Estimations place the number of tropical freshwater shrimp traded between 12 and 17 species, varying between countries, all within the genera *Atyopsis*, *Atya*, *Arachnochium*, *Caridina* and *Neocaridina* (Liptak and Vitázková, 2015). Across the marine ornamental industry, the trade of invertebrates (shrimp, hermit crabs, large polyp stony corals, snails and sea slugs) dominates the market, with 2.6× more invertebrates sold than fish (Watson *et al.*, 2023). In the marine market, a handful of species within the genus *Lysmata* and *Stenopus* constitute the majority of shrimp traded (Baeza and Behringer, 2017; Calado *et al.*, 2017). The most iconic include *L. amboinensis* (cleaner shrimp), *L. debelius* (fire shrimp), *S. hispidus* (boxing shrimp) and *L. boggessi* (peppermint shrimp) (Baeza and Behringer, 2017; Calado *et al.*, 2017), as well as *Hymenocera picta* (Harlequin shrimp) and *Thor amboinensis* (sexy shrimp) (Calado, 2020), (Fig. 1). Although, realised numbers of species may be significantly higher as many species are traded under the same common names, e.g. *L. bogessi* and *L. vittate* are both traded as ‘peppermint shrimp’. Whilst this is not an issue when scientific names are used, use of common names can lead to the misidentification, and so, misapplication of information for consumers. The dearth of biological and ecophysiological studies addressing reproductive biology and larval development, combined with difficulties of achieving a successful and sustained brood stock, has hindered the progress of marine ornamental shrimp culture, in contrast to that of their freshwater counterparts (Calado, 2008), leading to a greater reliance on wild collection.

### Transportation of tropical marine and freshwater ornamental species—current practice

For all aquaculture species, international transport is the engine that supports their global trade. This requires careful packaging for the live exportation and importation of species. Shipping costs can be substantial (Lim *et al.*, 2003; Paterson *et al.*, 2003); hence, it is in the best interest of importers and exporters to transport animals in ways which minimize shipping costs, while maximizing survival (Lim *et al.*, 2003; Harmon, 2009). All aspects of ornamental transportation aim to provide the necessary elements for survival, while reducing stress to the animal (Paterson *et al.*, 2003; Harmon, 2009). Standard methods for packaging ornamental aquatic species for international transport typically involve a closed system comprising two layers of polyethylene bags filled with water, inflated with air or oxygen (O_2_) and sealed (Correia and Rodrigues, 2017). Marine fish and invertebrates are most commonly bagged individually (Chuan *et al.*, 2010). By contrast, packaging practice for tropical freshwater species can involve higher loading densities (Lim *et al.*, 2003; Mandal *et al.*, 2010), although exceptions are common for species considered to be aggressive or those with delicate fins (Chuan *et al.*, 2010). For fish, loading densities [biomass of fish (g) per unit volume of water (L)] in packaging should be determined by the size, body mass and species of the fish, volume of the transport water and transport time (period from packing to unpacking) (Ramachandran, 2002; Lim *et al.*, 2003; Mandal *et al.*, 2010). Approximately 1 kg of fish can be placed per 10 l of water (Ramachandran, 2002; Belema *et al.*, 2017), but in reality stocking densities vary massively. Animals are then, most commonly, shipped internationally by air freight (Watson and Martinez, 2010; Correia and Rodrigues, 2017).

**Figure 2:**
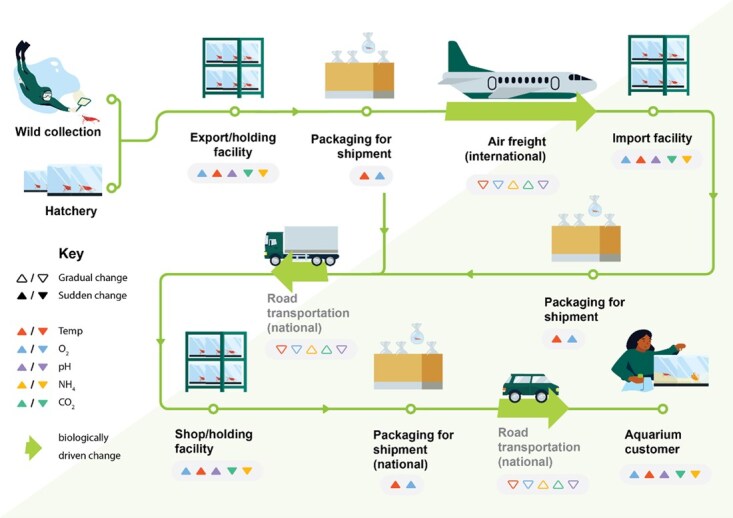
Schematic illustration of the typical journey marine and freshwater ornamental animals experience during their trade and transportation within the aquarium trade, from wild capture/hatchery production to the aquarium customer. Key physico-chemical parameter changes during each step of this journey are indicated by coloured arrows; arrows pointing upwards indicate an increase, while arrows pointing down indicate a decrease in that specific parameter. Filled arrows are used to show a ‘sudden’ environmental change caused by the physical movement or relocation of an animal to a new environment by human intervention, e.g. the movement of an animal from its tank to a plastic bag or vice versa. Hollow arrows indicate a ‘gradual’ change in physico-chemical parameters, in absence of human interference, e.g. gradual increase in NH_3_ in a sealed bag during transport due to biological processes, with the magnitude of change dependant on duration of that stage and biological activity. Figure by Hannah Whitman, Science Animation Studio.

The successful shipment of livestock relies on animals arriving in good health, with minimal mortalities. These outcomes are influenced by seemingly minor variations in shipping methods, changes in water quality and the physiological tolerance and capacities of the animals being transported (Chow *et al.*, 1994; Paterson *et al.*, 2003; Harmon, 2009). Throughout the process of collection, acclimation and international transport, animals may encounter significant stress, discomfort and abrasion, which can compromise their health and lead to mortality. Water quality can undergo changes during transport, exposing animals to elevated levels of ammonia (NH_3_), carbon dioxide (CO_2_), pH and temperature instability, as well as reduced dissolved O_2_ (Berka, 1986; Holmes *et al.*, 1999; Golombieski *et al.*, 2003; Harrison, 2016). A typical journey, together with the physico-chemical changes ornamentals may be exposed to during their trade, is depicted in Fig. 2.

The duration of transport governs the factors that impose the greatest mortality risk. For fish, pH reduction is the primary concern during short journeys (≤8 h), whereas NH_3_ excretion becomes the major risk factor during long journeys (>8 h) (Sampaio and Freire, 2016; Cerreta *et al.*, 2020). Similar definitive information on the key environmental factors governing survival during transport have yet to be determined for species of ornamental invertebrates. Minimizing mortality associated with the trade and transportation of ornamental species is a major challenge in the industry, which is also influenced by the accessibility of resources and capital restrictions of exporters (Harmon, 2009). Mortality rates vary widely, ranging from minimal levels (<5%) to as high as (but occurring rarely) 80% in each shipment (Johannes and Riepen, 1995; Sadovy and Vincent, 2002). Nevertheless, there is little knowledge on the precise changes in water quality during commercial transport, and limited understanding of the cumulative effects of repeated transport, and exposure to water of varying quality, on the health and survival of many ornamental species (Vanderzwalmen *et al.*, 2021). The practices currently employed in the shipping of marine ornamentals have arisen organically, including the various mitigations employed during their trade, when considering the different physico-chemical challenges the ornamentals face. In the absence of species-specific data, water-quality management during trade and transport of ornamentals typically relies on broad, generalized ‘standards’. These standards are often based on the assumption that ornamental species are uniformly sensitive to physico-chemical changes. While such a precautionary approach can reduce the risk of acute stress and mortality, it could also result in unnecessarily conservative and costly practices. In some cases, generic standards and thresholds may not reflect the optimal requirements of individual species potentially exposing animals to suboptimal conditions that may compromise long-term health.

Given what has gone before, the following sections examine the principle physico-chemical parameters that are subject to fluctuation during transport and review the mitigation techniques currently employed by stakeholders to manage them.

#### Temperature

Drawing upon empirical practice, it has long been believed that different ornamental species have a preferred temperature range of 24–27°C for tropical marine species (Hunziker, 2005; Koike, 2013; Correia and Rodrigues, 2017; Velasco-Blanco *et al.*, 2019) and 22–27°C for tropical freshwater species (Ramshorst, 1980). Therefore, it is widely considered that a constant, ‘optimal’, temperature should be maintained during transport; however, this is not entirely feasible during the international transportation of livestock. Instead, it is common to find temperatures gradually decline over the period of transportation (Mandal *et al.*, 2010; Vanderzwalmen *et al.*, 2021). In particular, during air freight temperature profiles can significantly change due to factors, such as variation in ambient temperatures and loading and unloading procedures (Lorenzon *et al.*, 2008; Watson and Martinez, 2010; Holroyd *et al*.*,* unpublished observations), altering water temperature profiles. This is most pronounced when tropical species are transported to temperate climates (Ramachandran, 2002). In most cases, rapid changes in temperature are ameliorated through the adequate insulation of the transport container with materials such as plastic foam or polystyrene, together with reflective material (Ramachandran, 2002). Thermal heat packs may also be placed within the container, and individuals may be acclimated to lower temperatures prior to shipment (Correia and Rodrigues, 2017). High temperatures, exceeding tolerance, can also be fatal to animals, so avoiding water temperatures rising above starting temperatures is advised (Ramachandran, 2002; Mandal *et al.*, 2010). Changes to temperature are advised to be kept to a minimum (<5°C) (Mandal *et al.*, 2010); however, literature lacks precise descriptions of safe maximal and minimal values, as well as safe rates of change. On arrival at a more permanent holding facility, any effects of suboptimal temperature exposure are usually remedied through gradually raising or lowering temperatures back to normal maintenance temperatures (Ramachandran, 2002). Exactly how optimal these current practices are in maintaining species health has not received any systematic appraisal.

#### Dissolved 0_2_

Ensuring sufficient O_2_ availability in aquaria and during transport is essential to the survival of any animal (Mandal *et al.*, 2010). While well-maintained aquariums seldom experience O_2_ shortages (termed hypoxia) or the complete absence of O_2_ (anoxia), O_2_ decline remains a significant risk during the transportation of livestock, necessitating careful management (Lim *et al.*, 2003; Mandal *et al.*, 2010; Sampaio *et al.*, 2019; Vanderzwalmen *et al.*, 2021).

In ornamental fish, several factors have been shown to influence the rate of O_2_ consumption (MO_2_) during transport, including the effects of environmental stressors such as temperature, salinity, NH_3_ and pH, alongside mechanical and handling stress, body mass and feeding level (Mandal *et al.*, 2010; Zarantoniello *et al.*, 2021; Fang *et al.*, 2023). Consequently, MO_2_ during transport is typically elevated (Mandal *et al.*, 2010; Zarantoniello *et al.*, 2021; Fang *et al.*, 2023), leading to faster rates of decline in the partial pressure of O_2_ (*P*O_2_) and in very severe cases, bagged animals may be exposed to severely hypoxic conditions. To compensate for reductions in *P*O_2_ during transport, either air or pure O_2_ is introduced into the bags before sealing (Lim *et al.*, 2003; Correia and Rodrigues, 2017), and animals may be starved for 12–24 h prior to shipment to reduce metabolic processes (Mandal *et al.*, 2010). Water to O_2_/air volume ratios are usually maintained around 20–35% water to 65–80% headspace, depending on the size of the animal and bag volume (Cole *et al.*, 1999). Yet, the efficacy of these mitigation methods in preserving the health and survival of different ornamental species during transport remains unexamined and uncertain. Furthermore, the introduction of O_2_ into transportation bags prior to shipment (Lim *et al.*, 2003; Correia and Rodrigues, 2017) could result in exposure to acute hyperoxia (heightened levels of O_2_), which could disrupt normal metabolic and respiratory regulation. This highlights the need for more detailed species-specific understanding of how O₂ dynamics change during transportation and, in turn, how these changes affect ornamental species’ physiology.

#### Carbonate chemistry (CO_2_ and pH)

CO_2_ not only dissolves in sea water but also chemically reacts with elements already present in that water, resulting in, among other things, increases in the partial pressure of CO_2_ (*P*CO_2_) (hypercapnia), increases in bicarbonate and carbonate concentrations and reductions in pH. Overall increased *P*CO_2_ plays a significant role in shaping water carbonate chemistry, and this in turn affects the extracellular acid–base balance of aquatic animals. CO_2_ accumulates in closed aquatic systems, such as containers or bags during transport, particularly when stocking density is high (Mandal *et al.*, 2010), affecting the health and survival of animals during transport (Melzner *et al.*, 2009; Thomas *et al.*, 2022). CO_2_ itself eventually becomes toxic, when present in sufficient concentrations, and compromises the ability of animals to extract O_2_ from water (Ramachandran, 2002). When animals are transported in poorly aerated water, the risk from changes in carbonate chemistry increases (Ramachandran, 2002). Concentrations of CO_2_ in excess of 40 mg·l^−1^ can be fatal (Mandal *et al.*, 2010). On arrival, indications of toxicity, including the loss of righting, can be addressed through aeration of the water (Ramachandran, 2002), although this may not always result in the successful recovery of individuals. Therefore, research is required to elucidate the possible impacts of altered carbonate chemistry, and how it affects health, as well as effective mitigation of adverse conditions, during transport.

#### Salinity

Many in the trade think, and would say, that maintaining osmotic and ionic balance (osmo- and iono-regulation) between the extracellular fluids (haemolymph or blood) and the surrounding medium is a requirement for good health and, therefore, requires provision of appropriate environmental salinities (Mantel and Farmer, 1983; Freire *et al.*, 2008; Rahi *et al.*, 2020). In tropical marine ornamental aquariums, water salinity is advised to be kept between *S* = 34–35 (Spotte, 1993; Skomal, 2006; Carter, 2023), whereas trace amounts (*S* = 0–0.1) are advised for freshwater species (Ramshorst, 1980; Gay, 2005). Fluctuations in salinity arise due to variations in the input of freshwater and the loss of water through evaporation (Chung *et al.*, 2025; Davila *et al.*, 2025). When managed correctly, salinity should not be an issue in well maintained aquaria (Hunziker, 2005; Koike, 2013). However, particularly in saltwater aquaria, the reality of maintaining a constant salinity can be a challenge due to the constant evaporation of water, necessitating freshwater top-ups (Spotte, 1993; Skomal, 2006; Carter, 2023). In many cases, efforts are made to ensure the salinity of the transportation medium is kept identical to the medium in which animals are maintained to minimize stress and mortality (Mandal *et al.*, 2010). Whether this parameter is well maintained during the period of transport is largely unknown, as there is little quantitative documentation of how salinity changes over time during the international exportation and importation of livestock. However, as livestock are sealed in polyethylene bags (Correia and Rodrigues, 2017), it is assumed that evaporation will not alter salinity.

#### NH_3_ and nitrogenous waste 

Nitrogenous waste is produced via protein catabolism and the decomposition of excreted faecal waste and unconsumed feed (Randall and Tsui, 2002; Chang *et al.*, 2015). These are mainly in the form of dissolved inorganic nitrogen ions ammonium, nitrite and nitrate (Romano and Zeng, 2013). When ionized, they constitute the most common forms of dissolved inorganic nitrogen in aquatic ecosystems. In confined systems such as aquaria, and during transport, metabolic wastes can accumulate rapidly, especially with high loading densities (Lim *et al.*, 2003). In the ornamental trade, small water volumes and high stocking densities exacerbate this challenge, leading to compromised health, growth and survival (Lin and Chen, 2003; de Lourdes Cobo *et al.*, 2014).

The ideal acceptable level of NH_3_ is considered to be 0 ppm, with mortality thought to occur when toxic unionized NH_3_ surpasses tolerable levels (<0.25 ppm) (Lin and Chen, 2003; de Lourdes Cobo *et al.*, 2014). These operational levels seem to be based on practice rather than rigorous empirical work. Elevated NH_3_ during transportation is cited as the key driver of fish mortality during long journeys (>8 h) (Sampaio and Freire, 2016), with mortality commonly reported around elevated levels of 1 ppm (Mandal *et al.*, 2010). Toxic concentrations of NH_3_ have not been determined for most species of ornamental invertebrates, yet changes in *P*O_2_, temperatures and pH are known to affect NH_3_ toxicity. A higher pH, reduced temperature and reduced *P*O_2_ results in greater ionization, increasing the toxicity of NH_3_ (Ramachandran, 2002; Mandal *et al.*, 2010). For example, a shift from pH 7 to 8 produces a 10-fold increase in the quantity of unionized NH_3_ (Ramachandran, 2002).

To mitigate the deterioration of water quality, resulting from the accumulation of metabolic wastes, numerous techniques are employed, including the starvation of animals prior to packaging (Phillips and Brockway, 1954; Nemoto, 1957), lowering temperatures of transport water (Phillips and Brockway, 1954; Nemoto, 1957; Teo and Chen, 1993), use of buffers (McFarland and Norris, 1958; Teo *et al.*, 1989), e.g. sodium bicarbonate and anaesthetics (Teo *et al.*, 1989; Teo and Chen, 1993), e.g. eugenol, for tropical freshwater animals. However, the efficacy of these techniques in mitigating the toxic effects of NH₃ and nitrogenous waste during trade and transportation is poorly understood. Unnecessary or improperly applied interventions may impose additional physiological stress, potentially exacerbating health and mortality risks. Without a comprehensive understanding of how NH₃ and nitrogenous waste affects the physiology of ornamental species, it is challenging to predict the extent to which these ions contribute to declines in health, mortality during trade and transportation or whether current mitigation practices are necessary, effective and economically justified. Robust species-specific empirical research is, therefore, essential to inform and refine these practices.

### Mechanistic knowledge gaps in ornamental shrimp

Understanding of transport-related stress in ornamental shrimp (see Transportation of tropical marine and freshwater ornamental species—current practice section) is constrained by a limited mechanistic understanding of how these taxa physiologically respond to environmental drivers. Current knowledge of caridean physiological responses to environmental variation is, in large, based on studies of penaeid shrimp and other aquaculture taxa.

Across these taxa, variation in key physico-chemical parameters, including temperature, salinity, dissolved O_2_, carbonate chemistry and nitrogenous waste elicits coordinated physiological responses across different hierarchical (cellular, tissue and whole-animal) levels. For example, in penaeid shrimp, including *Litopenaeus vannamei*, *Penaeus kerathurus*, *Penaeus japonicus* and *Metapenaeus monoceros*, osmoregulation is primarily achieved through isosmotic extracellular ion exchange across gill epithelia, and intracellular solute adjustments in organic osmolytes that maintain haemolymph ionic and osmotic homeostasis under fluctuating salinity (Huang *et al.*, 2026). Acid–base balance under changing pH conditions is regulated through branchial ion transport and bicarbonate buffering mechanisms, involving coordinated activity of gill epithelial transport systems that integrate ion exchange processes with NH_3_ handling to maintain broader osmophysiological homeostasis (Fehsenfeld and Weihrauch, 2017). These physiological mechanisms have been extensively characterized and reviewed in wider literature.

In contrast, equivalent mechanistic understanding for ornamental shrimp is poor. Although, to some extent, penaeid species provide a useful comparative framework for inferring general crustacean physiology, whether or not that understanding generalizes to ornamental taxa is uncertain. Ornamental shrimp comprise ecologically distinct reef-associated taxa (Calado, 2008) with life histories not represented in penaeid models and are subject to distinct environmental and operational pressures associated with their trade (Wabnitz *et al.*, 2003; Rhyne *et al.*, 2017).

Many commercially important aquaculture species were adopted for production because they possess relatively broad environmental tolerances and can maintain growth under variable or suboptimal conditions, contributing to their economic viability. For example, *L. vannamei* is a commercially favoured species due to biological attributes, including its euryhaline capacity and tolerance to a broad range of temperatures (Emerenciano *et al.*, 2022). Consequently, the physiological capacities and mechanistic bases underlying these responses in these taxa likely differ substantially from those of tropical ornamental shrimp. These differences are likely further amplified by different domestication histories. Unlike penaeid aquaculture species that have undergone decades of selective breeding and optimization for intensive production environments (Ren *et al.*, 2025), many ornamental shrimp entering the aquarium trade lack any domestication history. Indeed, many ornamental species, particularly tropical marine taxa, are primarily wild collected (Dominguez and Botella, 2014) and, therefore, represent populations naive to captive husbandry conditions. As such, while the mechanistic understanding derived from penaeids provides an important foundation for hypothesis generation and comparative interpretation, these frameworks may not fully capture the physiological responses and elucidate the optimal husbandry requirements of ornamental taxa without targeted validation.

This lack of mechanistic understanding limits the development and parameterization of ecophysiological frameworks for ornamental taxa and constrains evidence-based optimization of husbandry and transport practices. Addressing these knowledge gaps are both a scientific and conservation priority, as improved mechanistic understanding would support the refinement and optimization of captive husbandry protocols, enhance survival and reproductive success and, ultimately, reduce reliance on wild collection of ornamental species.

### Current practice—where do we go from here?

Overall, the success of aquatic ornamental trades rests on the appropriate execution of effective practices to ensure sufficient oxygenation, reduce the build-up and impact of metabolic waste products and, generally, avoid the development of an unhealthy environment during the transportation of tropical marine and freshwater species, that would otherwise impact the appearance and underlying health of the animal. This practice has evolved organically, over many years, but has rarely been informed by rigorous scientific research. The extent to which current practices, and thereby the health of transported livestock may be improved by integrating scientific, specifically ecophysiological, knowledge of the responses of aquatic animals, is as yet unexplored.

Tropical (marine and freshwater) ornamental shrimp present an ideal exemplar group through which to explore this, considering their immense popularity, coupled with the fact that ecophysiological approaches are not yet widely integrated in their management during their trade. The combination of high demand, critical industry relevance and substantial knowledge gaps positions ornamental shrimp as an ideal case study for demonstrating the benefits of applying ecophysiology to ornamental aquaculture. The main aim of this paper is therefore, to demonstrate how ecophysiological approaches can be applied to improve the health and survival outcomes of ornamental shrimp during trade and transport. While shrimp serve as the primary focus here, the principles and frameworks outlined within this paper have wider applicability across ornamental aquarium species, offering a pathway towards more systematic, evidence-based strategies to enhance health, reduce mortality and strengthen sustainability within the global aquarium trade. Specifically, we (i) show how ecophysiology can be used to inform an operational definition of health, using Hatch’s (1962) scheme as originally applied to occupational health, (ii) consider how ecophysiological approaches could be applied to ornamental management to improve health outcomes for traded animals, (iii) review current knowledge of how key physico-chemical parameters (temperature, dissolved O_2_, salinity, carbonate chemistry and nitrogenous waste) affect the physiology of tropical ornamental shrimp and (iv) identify knowledge gaps and research opportunities that could inform evidence-based management of these species.

## Health

### Physiological approaches to ‘health’

Although ‘health’ is a commonly used term, few explicit and consistent definitions exist for what it constitutes, particularly in animal management. Traditional definitions equate health with the absence of disease or injury, implying that it is a passive state. More contemporary perspectives, however, view health as a dynamic condition encompassing complete functional wellbeing (World Health Organisation, 1948), recognizing that it extends beyond the mere absence of disease and is not a dichotomous state (Arah, 2009; Nordenfelt, 2011; Ayres, 2020). Health is increasingly viewed as the ability to capitalize on full functionality, satisfy needs and adapt to, or manage within, changing environments (Eriksson and Lindström, 2008; Wittrock *et al.*, 2019).

Historically, veterinary science approaches to health focused primarily on disease-centric models, pathophysiology and biological functioning (Nordenfelt, 2011; Wittrock *et al.*, 2019). More holistic perspectives now embrace a broader array of factors when assessing the health and condition of an animal including (i) ‘normal’ appearance and behaviour (normality); (ii) biological function; (iii) physical and psychological well-being; (iv) productivity (reproduction) and finally, (v) physiological homeostasis, such as heart rate (Gunnarsson, 2006). Central to this growing understanding is the recognition of physiological homeostasis, defined as an organism’s ability to maintain internal stability while adjusting to changing external conditions (Cannon, 1929), emerging as a foundational concept to understand health.

Homeostasis reflects a state of dynamic equilibrium allowing some degree of independence from changing environmental conditions (Billman, 2013, 2020; Libretti and Puckett, 2023). Homeostatic control mechanisms facilitate physiological adjustments to maintain internal equilibrium, operating at various hierarchical levels within the body from molecular, cellular, tissue and organ, through to the whole animal (Ayres, 2020). Importantly, the health of an animal is said to be the end product of homeostatic regulation, reflecting a culmination of these dynamic regulatory processes (Billman, 2020). As such, meaningful assessments of health require an appreciation of this fundamental concept.

**Figure 3:**
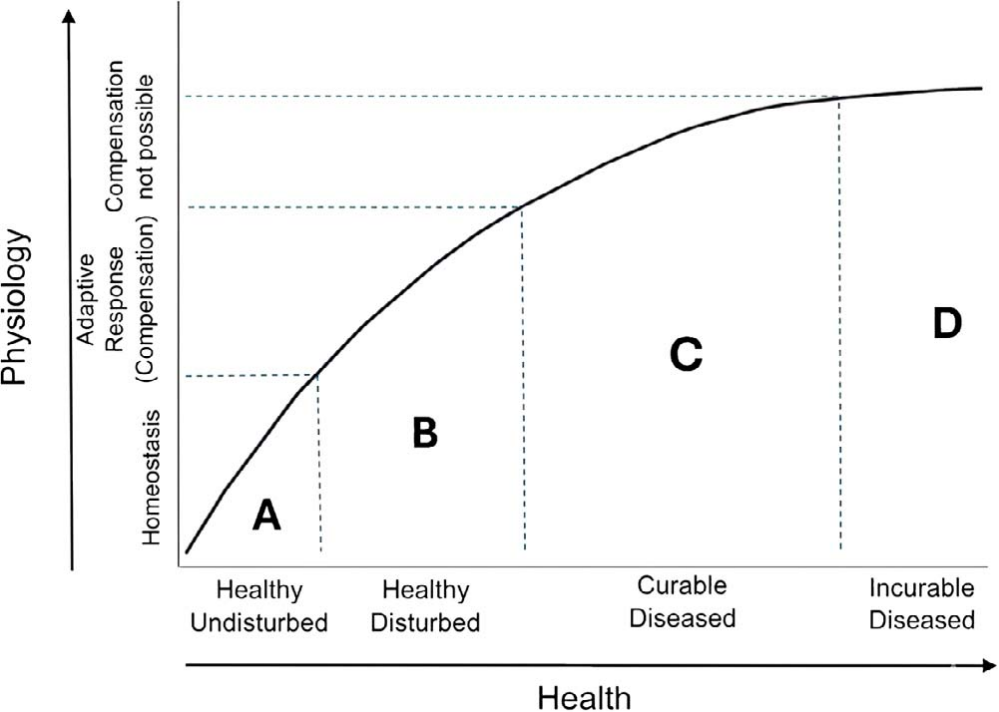
The dynamic relationship between health and physiology. The health status is indicated by the organism’s capacity for physiological compensation in response to environmental stimuli (disturbance). The continuum explicitly incorporates Hatch’s (1962) distinction between ‘curable diseased’ and ‘incurable diseased’ states, representing progressive loss of physiological capacity and recovery potential. Modified from Hatch (1962).

While homeostasis provides a foundational framework for understanding physiological stability through the maintenance of regulated physiological ‘set-points’, alternative frameworks such as allostasis (achieving stability though change) (Sterling and Eyer, 1988), highlight that organisms may alter these ‘defended’ physiological levels in response to environmental demand (). This approach considers allostatic load, the cumulative bioenergetics cost of maintaining chronically displaced physiological states, which can compromise the capacity to respond to future stressors. However, in invertebrate physiology, with some notable exceptions (e.g. Freire *et al.*, 2020), allostatic theory remains largely conceptual; the absence of validated metrics and clearly defined regulatory thresholds limits its operationalization (Schutle, 2014). Therefore, we adopt a homeostasis-centred approach, using Hatch’s (1962) framework (Fig. 3) to guide discussion of the operationalization of health within ornamental management.

A healthy animal, as described by Hatch (1962) (Fig. 3), demonstrates the capacity for physiological compensation and recovery in response to environmental stimuli. This idea refers to maintaining physiological homeostasis (‘vigour’) or returning to a ‘normal’ state, whereby some degree of physiological plasticity enables the animal to ameliorate internal changes brought about in a changing environment. Therefore, Hatch’s (1962) approach to health is the one we utilize within this paper. Defined as such, this positions health along a continuum, centred on physiological homeostasis, emphasizing adaptive capacity rather than absence of infectious and non-infectious disease. Hence, we propose that health fundamentally reflects an animals’ capacity to adaptively respond to stress through physiological adjustments, minimizing the costs of these adjustments and so, reducing the risk of adverse or chronic effects on future adaptability. This adaptive framework is particularly critical for aquatic animals, whose health and survival are intimately linked to variable environmental conditions (Chow *et al.*, 1994; Paterson *et al.*, 2003; Harmon, 2009), and by extension, management practices employed in sectors, such as ornamental aquarium trade.

Despite the recognized value of integrating physiology into animal health assessment, operationalizing this approach remains challenging. Individual variability in physiological responses (Cockrem, 2022) and ethical constraints limit both our understanding of animal physiology (Ayres, 2020; Kiani *et al.*, 2022) and its translation into practical applications. The literature provides limited insight into the relationship between physiological homeostasis and environmental stressors, with most studies only inferring potential health implications or mortality under chronic, repetitive or severe exposures. Despite efforts to advocate for the use of integrative approaches to assess animal health, disease or pathogen-centric models continue to dominate animal health evaluations (Lerner and Berg, 2017; Ayres, 2020; Stentiford *et al.*, 2020; Picard, 2022), particularly for aquatic livestock, where mechanistic frameworks for evaluating health are scarce. No established framework guides the adoption of ecophysiological approaches, highlighting the need for a move towards standardized ecophysiology-based protocols and best practices to support more comprehensive and predictive assessments of animal health.

### Current assessments of health in the aquatic ornamental industry

Within the ornamental industry, many sellers diligently strive to obtain and maintain livestock in optimal health, making regular assessment of livestock health an integral part of daily routines. The maintenance of livestock health is supported through good water quality management, appropriate nutrition and husbandry practices designed to minimize physiological disturbance. Water quality parameters are typically maintained within broadly acceptable ranges, guided largely by empirical experience or generalized husbandry recommendations. Diet and feeding management are similarly important for sustaining normal growth, immune function and overall condition (Pohlenz and Gatlin, 2014).

Health assessments in this sector typically involve visual morphological and behavioural evaluations of livestock (Stevens *et al.*, 2017), which provide non-invasive indicators of health. Any deviations from established norms, such as changes to feeding patterns (lethargy), increased ventilation, ‘flashing’ against objects or alterations in colouration (hyperpigmentation) are interpreted as indicators of underlying stress. Subsequently, appropriate measures are taken to address the identified cause and improve the organism’s health (Hargreaves, 2002; Pasnik *et al.*, 2010). For the experienced aquarist, these assessments allow for swift diagnosis and timely treatment. However, interpretation of these metrics can also be challenging and are subject to the experience of the observer. Many behaviours remain poorly characterized in scientific literature, and current knowledge is subjectively biased towards vertebrates, given their more distinct physical and behavioural characteristics (Stevens *et al.*, 2017).

In addition, more invasive techniques can be employed to manage disease and pathogen outbreaks in ornamental livestock. These include freshwater or saltwater dips (animal is submerged) (Buchmann, 2022), chemical treatments such as copper and broad-spectrum antibiotics (e.g. chloramphenicol, nitrofurans and erythromycin) and disinfectants (e.g. formalin, malachite green, potassium permanganate and methylene blue) (Chanda *et al.*, 2011; Yanong and Lewbart, 2024), which are commonly adopted by the industry. While a limited number of these treatments (e.g. certain antibiotics) can be safely applied to some ornamental invertebrates, many are primarily formulated for fish and can be toxic to invertebrates. Freshwater and saltwater dips, and copper-based treatments are lethal to invertebrates. As a result, invertebrate health management relies largely on quarantine, precise water quality control and the provision of a nutritionally appropriate diet, demonstrating the need for precise characterization of these components to optimize health outcomes for ornamental invertebrates.

Adopting a precautionary approach, strict biosecurity protocols are often employed for the arrival of new livestock within the ornamental trade, with quarantining often used to prevent the proliferation of pathogenic organisms (Monticini, 2010). For invertebrates, corals, sea anemones and live rock (calcareous substrate containing microbial and invertebrate communities) 30–60 days of quarantine is widely applied, as this is proposed to sufficiently interrupt the life cycle of parasites and prevent the spread of pathogens (Wildgoose, 2001). But in practice, quarantining periods vary due to the adoption of divergent monitoring methods by exporters, importers and retail establishments.

Regulatory oversight for the consignment of live aquatic animals similarly adopts a disease and pathogenic-centric focus. Importations of organisms from non-EU countries require an Official Veterinary health certificate confirming that animals have be inspected less than 72 h before loading and show no clinical signs of disease, lesions nor external parasites. Certification also requires that containers are appropriately labelled, disinfected and maintained under conditions that do not compromise animal health, and that documentation is enclosed that contain declarations of intended use and assurances that animals are not intended for destruction or slaughter for disease eradication are included (Centre for Environment, Fisheries and Aquaculture Science (CEFAS), 2023).

Documented frameworks of formal health assessments in ornamental species are limited. For ornamental fish, Floyd and Beleau (1986) outlines an Aquarium Fish Health Management programme, emphasizing four key factors: the fish, the water, the container and nutrition. Daily visual assessments of behaviour and feeding are advised to identify potentially stressed or ill-adapted individuals, complemented by a combination of invasive and non-invasive examination techniques, including light anaesthesia, gill and fin biopsies and faecal sampling. Water quality management is highlighted as particularly critical, given the direct influence of temperature, chemical composition and environmental stability on fish physiology and immunocompetence. This framework remains one of the few systematic health management programmes described in ornamental aquaculture, though developed for fish. Comparable frameworks for managing tropical invertebrate health are almost entirely absent. To our knowledge, the only published example is Maciaszek *et al.* (2018), who describe health criteria used during international ornamental tropical shrimp breeding competitions (genera of *Caridina* and *Neocaridina*)*.* Criteria primarily target the detection of epibionts and genetic defects (physical deformities), further highlighting the dominance of disease- and pathogen-focused approaches. Although health is increasingly recognized as more than the mere absence of disease (Eriksson and Lindström, 2008; Arah, 2009; Nordenfelt, 2011; Wittrock *et al.*, 2019), industry and regulatory mandates continue to predominantly rely on disease-centric methodologies, often equating disease with a clinically detectable infection. In practice, adverse health outcomes and mortality can result from both infectious and non-infectious causes. Even in the absence of detectable disease, animals can experience mortality due to physiological failure from unsuitable physico-chemical conditions, nutritional imbalance or cumulative metabolic strain. A disease-centric focus thus limits the identification of broader health determinants and the development of practical guidelines grounded in ecophysiological principles.

Consequently, there is a missed opportunity to comprehensively understand physiological drivers of health, and to optimize the health of traded ornamentals. Expanding research, development and application of ecophysiological knowledge could enhance the management of organismal health within the aquarium trade. Through the integration and diversification of health assessments, from sole disease and pathogenic-centric approaches, more systematic and comprehensive assessments of health can be undertaken. Accordingly, we now turn to examine how an ecophysiological approach can be applied to improve the management of health in ornamental species during trade and transportation.

## An ecophysiological approach to ornamental management

### What is ecophysiology and why is it important

Ecophysiology is the study of how organisms function and adapt to environmental variation through the measurement of physiological responses, and how these responses, in turn, influence survival, as well as broader ecological interactions and processes (Bennett, 1987; McNab, 2002; Lambers *et al.*, 2008; Sommer, 2024). By quantifying organismal responses to key physico-chemical parameters, such as temperature, dissolved oxygen (DO), salinity, carbonate chemistry and nitrogenous waste, ecophysiology provides a mechanistic framework that complements current practice for operationalizing health, moving beyond the absence of disease to encompass a more integrative, holistic approach to health.

Addressing the questions ‘how does this organism work?’ and ‘how is its physiology modulated by the environment?’, requires multidisciplinary approaches that integrate field, laboratory and natural experiments (Spicer, 2014). Modern molecular and omics-based techniques complement traditional whole-organism measurements, providing mechanistic insight from biomolecules (Norwood and Gomez, 1994) to whole organism-level responses (Pörtner *et al.*, 2006; Kuster *et al.*, 2011). Together, these approaches offer mechanistic and predictive insight into organismal function, resilience and vulnerability, making ecophysiology a powerful framework for assessing health in ornamental species. By linking physiological function directly to environmental tolerance, ecophysiology bridges the gap between basic biological understanding and practical management.

Ecophysiology provides the mechanistic resolution to account for species-specific physiological tolerances that are largely lacking with current industry practices, where environmental conditions are maintained within broadly acceptable ranges, guided largely by empirical experience. Behavioural and morphological assessments, also frequently used as reactive indicators of stress or declining health within commercial ornamental management, similarly, fail to capture the physiological and mechanistic drivers underlying the observed responses. The integration of ecophysiological frameworks, therefore, offers the potential to extend beyond empirical optimization by quantifying critical thresholds for key environmental variables at which sublethal exposure begins to compromise physiological function and health. This approach is particularly valuable for ornamental aquaculture, as seen above, due to traded animals routinely being exposed to fluctuating, and sometimes extreme, physico-chemical conditions during their trade and transport (see Transportation of tropical marine and freshwater ornamental species—current practice). Such mechanistic insight would support husbandry interventions that not only mitigate physiological stress and reduce mortality but also promote optimal physiological performance, and ultimately, health. In this manner, ecophysiology provides a proactive, evidence-based foundation for refining health management in ornamental aquaculture, facilitating a transition from anecdotal, reactionary maintenance towards more precision-based approaches.

Ecophysiology has already demonstrated its value across a range of applied fields by linking mechanistic understanding of organismal function to practical management outcomes. In conservation biology, it has been instrumental in identifying and predicting physiological thresholds that define species’ vulnerability to current or future environmental perturbation linked to climate change, enabling predictions of survival and distributions under future conditions (Pörtner and Farrell, 2008; Bozinovic and Hans-Otto, 2015; Penn and Deutsch, 2022). In aquaculture for food production, ecophysiological principles have been applied to optimize rearing systems and environmental parameters, maximizing growth and biomass production efficiency (Parker, 2011). These examples highlight the power of integrating physiological understanding into management frameworks and demonstrate the potential for similar widespread integration into the aquarium trade, where livestock health and survival underpin commercial success (Johnstone *et al.*, 2017).

While ecophysiology encompasses a wide range of techniques, below, we focus on a few illustrative approaches to demonstrate its practical utility in the aquarium industry. These examples show how mechanistic insight into physiological function can inform evidence-based decisions, improving health outcomes, survival and overall welfare standards. In doing so, these approaches subsequently enhance conservation and sustainability outcomes of the aquarium trade.

### How do we practice ecophysiology?

Direct measurement of organismal responses is ideal but often impractical. Instead, ecophysiologists commonly utilize correlated indirect measures of performance (proxies) (Miller and Stillman, 2012), such as heart rate, ventilation, haemolymph and tissue metabolites (Taylor and Spicer, 1987, 1989; Frederich and Pörtner, 2000; Ern *et al.*, 2014). These proxies provide snapshots of physiological function across different environmental conditions and form the foundation for more integrative analyses. However, interpreting physiological proxies is often challenging, as organismal responses reflect complex interactions among autecology, physiology, behaviour and environmental stimuli (Crespi *et al.*, 2013; Johnstone *et al.*, 2017). Metrics alone may not provide meaningful insight without the broader biological context necessary for interpretation (Peig and Green, 2010).

**Figure 4:**
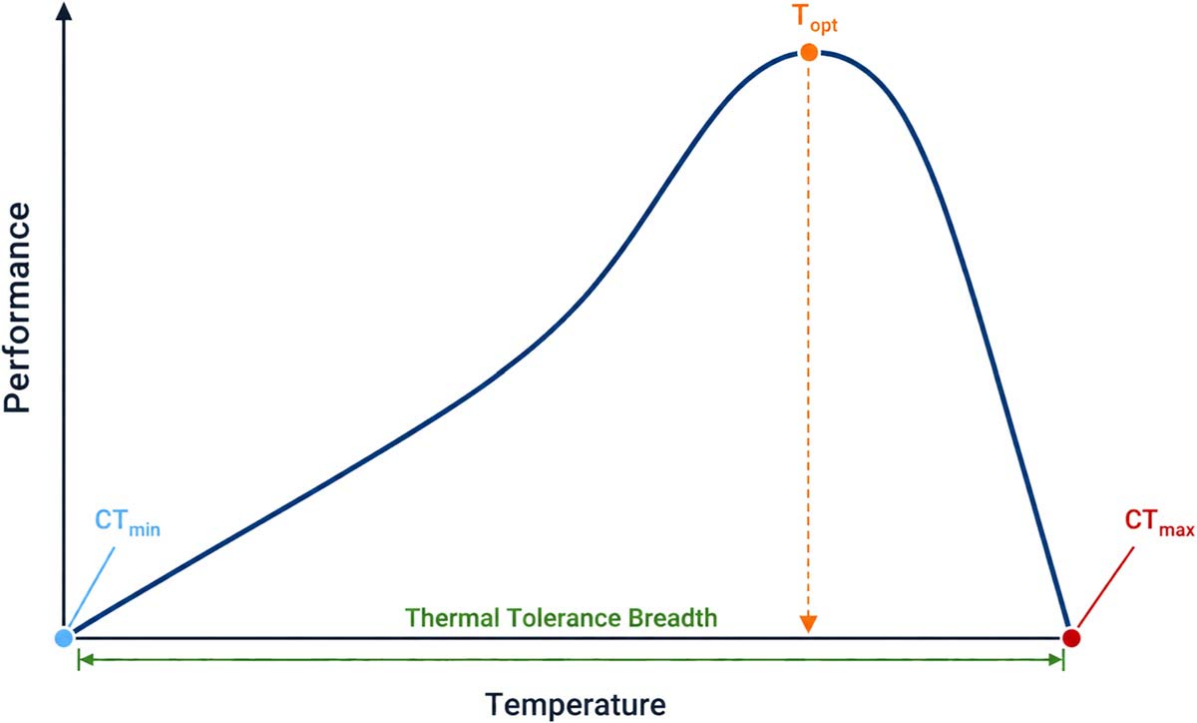
General standard thermal performance curve (TCP). Relationship between environmental temperature and a physiological rate of an ectotherm expressed as a TCP. TCP’s are typically characterized by an initial slow increase in performance with increasing temperatures, reaching maximum rate at the optimal temperature (*T*_opt_), followed by a rapid decrease until the point of complete cessation (CT_max_). CT_max_ and CT_min_ represent the *T*_A_ above and below which performance is at a minimum, and *T*_opt_ represents the *T*_A_ at which performance is maximum.

By systematically measuring these proxies across environmental gradients, physiological performance curves can be generated that show how functional capacity shifts under changing conditions and pinpoint ranges of metabolic instability, and scope for areas of unstable metabolic responses (Fig. 4) (Molnár *et al.*, 2017; Kellermann *et al.*, 2019; Little and Seebacher, 2021). Subsequently examining shifts in physiological rate(s), performance curves offer valuable insights into how an animal may navigate and compensate for changes in their environment, by adjusting underlying physiology (Little and Seebacher, 2021). These curves can be a useful tool to evaluate organismal tolerance and detect thresholds where physiological responses become compromised or unstable (Killen *et al.*, 2021), and thus, support interventions to safeguard animal health.

Having outlined the benefits of an ecophysiological approach, we review and assess how our current ecophysiological understanding, or perhaps lack thereof, could inform the practical management of ornamental aquarium livestock. For this, we utilize tropical ornamental shrimp as an example group to assess ecophysiological understanding in relation to key physico-chemical parameters (see An ecophysiological approach to ornamental management), identify gaps in understanding and discuss examples of how ecophysiological principles could be applied to address these gaps and subsequently improve husbandry practice. This approach highlights the potential of ecophysiology to underpin evidence-based management strategies, ultimately, demonstrating the need, benefits and opportunities this approach has.

## Physiological responses of marine and freshwater tropical ornamental shrimp to environmental challenges

Crustaceans, such as shrimp, are a valuable model for examining ecophysiological responses because their physiology is often tightly coupled to the physico-chemical conditions of their environment. Research has characterized the physiological responses of a variety of freshwater, brackish and saltwater shrimp to a range of ecologically relevant environmental challenges, but this body of knowledge is dominated by research on commercial food-based aquaculture species.

Despite, the commercial importance of the popular ornamental shrimp species (see ‘Ornamental Shrimp’), physiological research investigating how these species respond to environmental challenges is scarce. This lack of physiological and mechanistic insight highlights a critical disconnect between the commercial importance of these species and the empirical data required to inform and optimize their health management. As a result, current ecophysiological understanding on ornamental shrimp is largely extrapolated from other species, whilst the limited studies undertaken involving ornamental shrimp taxa have rarely been designed with aquarium trade in mind. Their findings may therefore only be incidentally relevant, when tested conditions haphazardly reflect those experienced during trade and transport. This gap highlights ornamental shrimp as a critical yet underexplored exemplar group for applying ecophysiological tools within the aquarium trade, where targeted research could generate data directly applicable to improving animal health, mortality and industry sustainability.

In this section, we synthesize available knowledge on the physiological responses of ornamental shrimp to key physico-chemical parameters relevant to aquarium trade and transport. Each factor is considered independently for clarity, although we recognize that in practice, animals typically experience multiple stressors simultaneously, i.e. ‘multi-stressors’ (Crain *et al.*, 2008; Jackson *et al.*, 2016). While understanding the role of multi-stressor interactions is crucial for accurately predicting organismal responses to environmental changes (Orr *et al.*, 2020), many of the interactions are not fully understood for ornamental shrimp and, therefore, are beyond the scope of this review. Instead, our aim is to consolidate baseline physiological knowledge, identify critical gaps in our understanding and highlight how targeted ecophysiological research could generate actionable insights to improve health management practices within the ornamental aquarium trade.

### Temperature

Species-specific data on the thermal physiology of ornamental shrimp is limited to a handful of studies, and the mechanisms shaping thermal sensitivity and tolerance are poorly understood (Somero, 2002; Hofmann and Todgham, 2010; Whiteley and Mackenzie, 2016). Despite frequent assumptions that tropical shrimp have narrow thermal tolerance ranges, data to substantiate these claims is lacking.

Rosa *et al*. (2014) examined the physiological response of two ornamental shrimps, cleaner shrimp (*L. amboinensis*) and peppermint shrimp (*Lysmata seticaudata*) to acute thermal warming, using cessation of the sensory antennules as the endpoint. Upper thermal limits (CT_max_) were 35.0°C (*L. seticaudata*) and 35.4°C (*L. amboinensis*), respectively, with acute warming increasing levels of heat-shock proteins (HSP70/HSC70), the upregulation of chaperone proteins and the concentration of the anaerobic end product L-lactate in muscle tissue. Malondialdehyde levels in *L. amboinensis* also increased, indicating that extreme cellular lipid peroxidation occurred. These findings showed that both species of ornamental shrimp can cope with acute increases in temperature (~11°C above acclimation), although the lack of attempted recovery and the absence of chronic exposure data limit conclusions about long-term resilience and health. While such extreme temperature changes are unlikely to occur in normal trade and transport, these results highlight the risk of potential physiological stress under suboptimal conditions and identify the need for targeted research to define optimal, suboptimal and lethal thermal limits to inform the management of water quality.

Further insights come from Holroyd *et al.* (unpublished observations), who assessed both upper and lower thermal limits in *L. amboinensis* using an industry-relevant ramping rate (0.3°C·h^−1^). Interestingly *L. amboinensis* exhibited a broad thermal tolerance (CT_max_ = 35.25°C and CT_min_ = 7.5°C). Cardiorespiratory system failure coincided with the upper thermal limit but could not explain the complete collapse of ventilation at critically low limits, as there was no evidence of recourse to anaerobic metabolism. These findings indicate that factors beyond O_2_ limitation likely play a role in determining the lower thermal limit. The study overall challenges anecdotal perceptions of extreme thermal sensitivity in cleaner shrimp and demonstrates considerable robustness to gradual temperature change within typical trade conditions. No comparable data could be found for other tropical ornamental shrimp, including the widely traded freshwater *Caridina* and *Neocaridina*. The absence of studies significantly constrains our understanding of the thermal biology of ornamental shrimp. Targeted ecophysiological research (see ‘Temperature’) is required to define the thermal thresholds and tolerances of ornamental shrimp, enabling the refinement of thermal management during trade and transport, and subsequently minimizing cases of poor health and mortality.

### Dissolved O_2_

To date, no studies have directly investigated the physiological responses of tropical ornamental shrimp to hypoxia or hyperoxia, leaving major gaps in both scientific understanding and practical guidance for their management.

The lack of reliable data presents a barrier to defining O_2_ requirements during trade. Optimal, suboptimal and lethal *P*O_2_ thresholds are undetermined for many ornamental species, and thus identification of species-specific MO₂ and hypoxic/hypertoxic tolerance, using indices such as critical O_2_ partial pressure, would allow the industry to deliver precise O_2_ supplementation in transport bags, minimize exposure to suboptimal conditions and reduce unnecessary use of O_2_, overall improving health outcomes and cost-efficiency for businesses. Beyond transport, such research would also improve our understanding of how ornamental shrimp may respond to changes in *P*O_2_ in natural habitats, providing dual benefits for both trade sustainability and conservation.

### Carbonate chemistry (*P*CO_2_ and pH)

Despite the commercial value of ornamental shrimp and the susceptibility of their natural habitat to rising acidification (Mollica *et al.*, 2018), few studies have investigated their physiological responses to pH change.

Primary insights on ornamental shrimp taxa from Taylor *et al.* (2015) found that exposure to *P*CO_2_-induced pH reduction altered exoskeleton mineralization and biophotonic properties in red rock shrimp (*Lysmata californica*), one of the several species traded under the common name peppermint shrimp and often mistaken for the true peppermint shrimp, *Lysmata wurdemmani*. pH reduction had no effect on exoskeletal growth (percentage increase in carapace length) or mean cuticle thickness but instead increased the proportion of calcium in the cuticle, resulting in a greater Ca:Mg ratio, and a 5-fold decrease in animal transparency. Prakash *et al.* (2022) reported that reduced pH increased the concentrations of organic acids (L-lactate and succinate) lower levels of antioxidants metabolites/enzymes, such as Ascorbic Acid (ACS) and Ascorbate Peroxidase (APX) , and increased glutathione *S*-transferase enzyme activity in glass shrimp (*Ancylocaris brevicarpalis*), indicating oxidative stress during exposure. The physiological processes of both species may be compromised by exposure to acidified conditions, posing a threat to their long-term survival across tropical reef environments. Although these studies offer valuable preliminary insights into how ornamental shrimp respond to carbonate chemistry changes, evidence remains scarce, leaving major gaps in our understanding. This lack of data presents a challenge for the ornamental trade, particularly during international transport, where shrimp are often sealed in plastic bags for extended periods (see Transportation of tropical marine and freshwater ornamental species—current practice above). However, the extent to which increased *P*CO₂ and associated declines in pH impact the health, physiology and mortality of tropical ornamental shrimp is not well understood. Expanding this knowledge is thus critical for identifying species-specific carbonate chemistry sensitivities and tolerances which can be applied to develop species-specific protocols, optimizing water quality and developing targeted mitigation strategies that minimize exposure to harmful, suboptimal or lethal conditions during trade and transport.

### Salinity

Despite the importance of salinity to crustacean physiology, the osmoregulatory capacity of ornamental shrimp remains largely uncharacterized.

In one of the few studies on the effects of salinity in ornamentals, Sandifer (1973) examined the effects of salinity and temperature on grass shrimp [*Palaemon* (as *Palaemonetes*) *vulgaris*] larval development, a popular species within the tropical freshwater trade. Low salinity (*S* = 5 and 10) reduced survival and slowed development, whereas survival exceeded 60% at higher salinities (10‰>) at both 20 and 25°C. These findings highlight the potential for salinity to directly affect survival and growth, particularly during early life stages. While this study provides some insight on the salinity effects of ornamental species, broader understanding remains almost non-existent. The physiological mechanisms underpinning osmoregulation, tolerance thresholds and the metabolic costs of salinity fluctuations remain largely uncharacterized for ornamental shrimp. Consequently, species-specific guidelines for optimal salinity management and prevention of osmotic stress in aquaria and during transport are absent.

### NH_3_ and nitrogenous waste

Currently, there are no empirical studies on the physiological responses to nitrogenous waste, nor tolerance thresholds, for ornamental shrimp.

The absence of species-specific data on ornamental shrimp highlights a critical knowledge gap, particularly given the assumed toxicity of these compounds and their tendency to accumulate in the water during transport (Lin *et al.*, 2003; de Lourdes Cobo *et al.*, 2014; Zhao *et al.*, 2020). Without a comprehensive understanding of how nitrogenous waste affects their physiology, including the mechanisms underlying tolerance, it remains unclear to what extent these ions contribute to stress, health deterioration and mortality during trade and transportation, and whether current mitigation strategies (see Transportation of tropical marine and freshwater ornamental species—current practice) are necessary, effective or economically justified (Phillips and Brockway, 1954; Nemoto, 1957; Teo and Chen, 1993; Ling *et al.*, 2000). Robust empirical research is, therefore, essential to fill this gap and refine best practices. Advancing knowledge of these mechanisms is important not only for selective breeding in food-based aquaculture but also for informing the selection and breeding of more resilient ornamental shrimp, whether from cultured lines or wild-capture populations.

## Conclusion and recommendations

This review has successfully addressed the aims outlined herein, demonstrating the utility of integrating ecophysiological approaches into health management of tropical ornamental shrimp and is, therefore, of value to researchers across ecophysiology, conservation biology and aquatic animal health, as well as to aquarium industry stakeholders responsible for husbandry and transport protocols.

The transition from reactive, disease and pathogen-centric health management towards a more integrated and rigorous evidence-informed model of health management is a biological necessity and logical progression for the global aquarium trade. While ornamental shrimp are used here as a focal case study to illustrate current ecophysiological knowledge gaps and their operational implications, the challenges identified are not taxon-specific. Rather, they reflect broader challenges within the trade. Accordingly, the principles and recommendations proposed here are derived from our assessment of ecophysiological knowledge gaps specific to ornamental shrimp but are intended to be applicable across all tropical aquarium taxa, many of which experience comparable knowledge deficits, and trade pressures.

Current husbandry protocols within the aquarium trade continue to rely on broadly applied standards that often overlook species-specific metabolic requirements and environmental tolerances. Such generalization may be pragmatic, but it risks exposing organisms to suboptimal conditions that compromise physiological performance and long-term health. By systematically quantifying the physico-chemical drivers of poor health and mortality, and the physiological pathways through which they affect function, management strategies can be refined to better align environmental conditions with biological requirements that maximize health outcomes for traded species. In doing so, management strategies can become both biologically informed and economically justified, reducing avoidable mortality while improving operational efficiency.

This review reveals a clear disparity; despite the substantial economic value and global scale of the aquarium trade, species-specific physiological and mechanistic understanding is absent for many commonly traded taxa. This knowledge gap constrains the progression of husbandry towards more rigorous evidence-based health management. Bridging this gap requires both recognition of the value that physiologically grounded frameworks can provide to the trade, and the integration of targeted research designed to address practical, trade-relevant challenges and inform husbandry practice. As the aquarium trade continues to evolve, long-term sustainability and optimization, ultimately, depends upon the continued refinement of its practices in alignment with the biology of the species it relies upon.

Improving health outcomes for traded animals may also, simultaneously, contribute to enhancing conservation outcomes and the ecological sustainability of the aquarium trade. Increased survivorship during trade and transport may lessen the need for mortality-driven extraction from wild populations to replace lost individuals. Improved physiological understanding can also support the refinement and optimization of captive breeding programmes, supporting longer-term population sustainability within the trade. Similar approaches have already been advocated for, and successfully integrated into, conservation and fisheries management. For example, Falco *et al.* (2022) demonstrated how physiological thresholds can inform stock assessments and conservation policy in the Mediterranean Sea, while Rodgers *et al.* (2019) used physiological data to inform the conservation and stock management of the green sturgeon (*Acipenser medirostris*). These examples illustrate how embedding mechanistic biological insight within management frameworks can directly support more sustainable and evidence-based practices. As we navigate the challenges posed by environmental change and global trade, ecophysiological approaches become imperative for informed decision-making, conservation efforts and to ensure the health and well-being of species in our care.

Thus, we conclude with the following recommendations:

(1) **Prioritize industry aligned research:** future studies should adopt experimental designs that mirror real-world conditions in trade to ensure data can be directly applied to husbandry protocols. Research should consider key needs and challenges in the aquarium trade to ensure that findings are actionable, relevant and can inform practical management decisions.
(2) **Develop and standardize ecophysiologically informed health indicators** for key ornamental species. Baseline data should be used to establish reference ranges for physiological biomarkers. While direct measurement of biomarkers may not be feasible during routine operations, this data will provide the mechanistic understanding driving widely used ‘proxies’ such as feeding behaviour, activity levels or colouration, allowing industry stakeholders to deploy more appropriate mitigation and management strategies.
(3) **Develop species-specific guidelines and/or protocols for species management:** Physiological thresholds should be translated into practical, accessible, low-resource guidelines for industry stakeholders, e.g. fishermen, exporters, importers, regulators, etc. across the supply chain. They should define optimal, safe and unsafe physico-chemical conditions, and provide actionable steps when environmental conditions or organismal health deviate away from the desired state. The development of such guidelines should arise through holistic, cooperative workings between industry stakeholders and scientific researchers to ensure relevance, feasibility and adoption.
(4) **Integrate ecophysiological knowledge into existing health protocols**, such as those set by regulatory bodies for imported livestock. By combining mechanistic insights with conventional assessments, industry stakeholders can implement practical monitoring systems that capture both environmental conditions and organismal responses. For example, supplementing existing biosecurity and veterinary checks with dynamic checklists/health scoring systems, and/or maintaining logs of environmental conditions and correlating these with health outcomes. Health scoring sheets could be used to record visual and behavioural observations (e.g. feeding behaviour, colour, etc.) alongside key water parameters (temperature, DO, etc.). Each parameter could be assigned a species-specific ‘green/yellow/red’ range derived from ecophysiological data, allowing identification of optimal and suboptimal conditions and proactive interventions (dosing and adjusting water quality, aeration, etc.) before health or survival is seriously compromised.
(5) **Establish long-term supply-chain monitoring programmes:** longitudinal monitoring of both water quality (e.g. temperature, DO, NH_3_ and salinity) and simple physiological indicators of stress at key stages, such as collection, holding, during transport, post transport and arrival. Tracking these parameters will build a clearer picture of how organisms respond to chronic or repeated stress during trade, providing the evidence needed to continuously refine and enhance best practices.

## Data Availability

No data were generated or analysed during this work.
